# Research on the method of identifying upper and lower limb coordinated movement intentions based on surface EMG signals

**DOI:** 10.3389/fbioe.2023.1349372

**Published:** 2024-01-10

**Authors:** Yongfei Feng, Long Yu, Fangyan Dong, Mingwei Zhong, Abigail Alexa Pop, Min Tang, Luigi Vladareanu

**Affiliations:** ^1^ Faculty of Mechanical Engineering and Mechanics, Ningbo University, Ningbo, China; ^2^ Academy for Engineering and Technology, Fudan University, Shanghai, China; ^3^ Robotics and Mechatronics Department, Institute of Solid Mechanics of the Romanian Academy, Bucharest, Romania; ^4^ Department of Neurological Rehabilitation, Ningbo Rehabilitation Hospital, Ningbo, China

**Keywords:** upper and lower limb coordinated movement, rehabilitation robot, motion intention recognition, SEMG signal, pattern classification

## Abstract

Rehabilitation robots have gained considerable focus in recent years, aiming to assist immobilized patients in regaining motor capabilities in their limbs. However, most current rehabilitation robots are designed specifically for either upper or lower limbs. This limits their ability to facilitate coordinated movement between upper and lower limbs and poses challenges in accurately identifying patients’ intentions for multi-limbs coordinated movement. This research presents a multi-postures upper and lower limb cooperative rehabilitation robot (U-LLCRR) to address this gap. Additionally, the study proposes a method that can be adjusted to accommodate multi-channel surface electromyographic (sEMG) signals. This method aims to accurately identify upper and lower limb coordinated movement intentions during rehabilitation training. By using genetic algorithms and dissimilarity evaluation, various features are optimized. The Sine-BWOA-LSSVM (SBL) classification model is developed using the improved Black Widow Optimization Algorithm (BWOA) to enhance the performance of the Least Squares Support Vector Machine (LSSVM) classifier. Discrete movement recognition studies are conducted to validate the exceptional precision of the SBL classification model in limb movement recognition, achieving an average accuracy of 92.87%. Ultimately, the U-LLCRR undergoes online testing to evaluate continuous motion, specifically the movements of “Marching in place with arm swinging”. The results show that the SBL classification model maintains high accuracy in recognizing continuous motion intentions, with an average identification rate of 89.25%. This indicates its potential usefulness in future rehabilitation robot-active training methods, which will be a promising tool for a wide range of applications in the fields of healthcare, sports, and beyond.

## 1 Introduction

According to statistics from the World Health Organization, approximately 15 million individuals worldwide suffer strokes annually, with over 80% of survivors experiencing motor function disorders (World Health Organization, 2023). Leveraging the human brain’s plasticity, precise and timely rehabilitation training facilitates patients in neural reorganization or compensation. This process stimulates the creation of new neural cells related to motor function, thus significantly enhancing the chances of survival and recovery of motor abilities (Rossini et al., 2003). Traditional hemiplegia rehabilitation, mainly conducted by physicians providing manual training assistance, presents challenges such as increased workload, reduced efficiency, and an unbalanced physician-to-patient ratio (Rossini et al., 2003). The incorporation of rehabilitation robots in limb motor function rehabilitation training emerges as a newfound prospect for individuals afflicted with paralysis (Zhang et al., 2017).

In the past decade, there has been continuous development of intelligent robots for limb rehabilitation, attracting extensive attention from scholars (Pérez-Bahena et al., 2023). However, the current focus of rehabilitation robots primarily centers on the limbs most affected in patients, with relatively less research dedicated to rehabilitation robots that address multi-limbs coordination and balance training systems (Mu et al., 2019). Recent research on upper limb rehabilitation robots has concentrated on the joint rehabilitation of upper limbs but lacks attention to lower limb rehabilitation needs (Durand et al., 2019; Xie et al., 2022; Wu et al., 2023). Similarly, recently developed lower limb rehabilitation robots can only provide training for the patients’ lower limbs (Han et al., 2019; Gao et al., 2022; Tian et al., 2022). However, relevant studies have shown that the movements of the upper and lower limbs are coupled and mutually influential during normal walking, with the normal swing of the upper limbs playing a crucial role in an individual’s walking (Dietz et al., 2002; Arya et al., 2019). Rehabilitation robots that can coordinate upper and lower limb training consider the comprehensive recovery of limb function. By applying theories of motor neuron coupling, these robots enhance the strength and coordination of both upper and lower limbs through specific task training (Fang et al., 2017; Huo et al., 2019) integrated coordinated upper limb swing functions into the Rowas rehabilitation robot, ensuring synchronous movement between the lower limbs and the upper limb shoulder joint, thereby achieving coordinated rehabilitation of both upper and lower limbs in patients (Huang et al., 2023). designed an exoskeleton-based upper and lower limb rehabilitation robot system, planning training trajectories for the hip, knee, and shoulder joints. Therefore, it is necessary to design a robot that coordinates upper and lower limb rehabilitation. This approach aims to induce and reorganize abnormal coupling symptoms in the motor nerves of stroke patients, thereby enhancing rehabilitation treatment for the balance and coordination of the patient’s limbs.

Clinical rehabilitation research suggests that tailoring rehabilitation training to the patient’s limb movement patterns enhances rehabilitation efficiency (Pichiorri et al., 2015; Song et al., 2023). sEMG signals, known for their non-invasiveness and operational simplicity, serve as a common tool to reflect human muscle activity, facilitating research in human motion classification (Wu et al., 2016). employed a LLE model to streamline algorithm complexity and utilized the ELM for the swift classification of upper limb movements involving the shoulder, elbow, and wrist (Shao et al., 2020). accurately identified movements of the shoulder, elbow, and wrist joints using a combined SVD-WDBN classification model (Hu et al., 2021). used a sEMG array sensor to collect electrical signals from the muscles of the wrist and successfully recognized discrete gestures and continuous movements. However, the current stage of sEMG signal pattern recognition primarily focuses on single areas such as the upper limbs or hands, lacking research on the recognition of coordinated movement intentions between upper and lower limbs. Therefore, there is a need to develop an algorithm for multi-limbs movement intention recognition based on multi-channel sEMG signals. This algorithm would adapt to training movements of U-LLCRR, to achieve the goal of human-machine interaction.

In this study, the research mainly focuses on developing a human upper and lower limb coordinated movement intention recognition method based on the developed U-LLCRR and sEMG signals. The meachnical structure and hardware control system of the U-LLCRR are designed. Based on the robot’s training mode, the study designs a recognition scheme for continuous motion, specifically the movements of ‘Marching in place with arm swinging’. The SBL classification model is developed, integrating various classification models to enhance the classification of the extracted features. This study establishes a foundation for subsequent research in human-machine interaction control.

## 2 Materials and methods

### 2.1 Mechanical structure design of U-LLCRR

**FIGURE 1:**
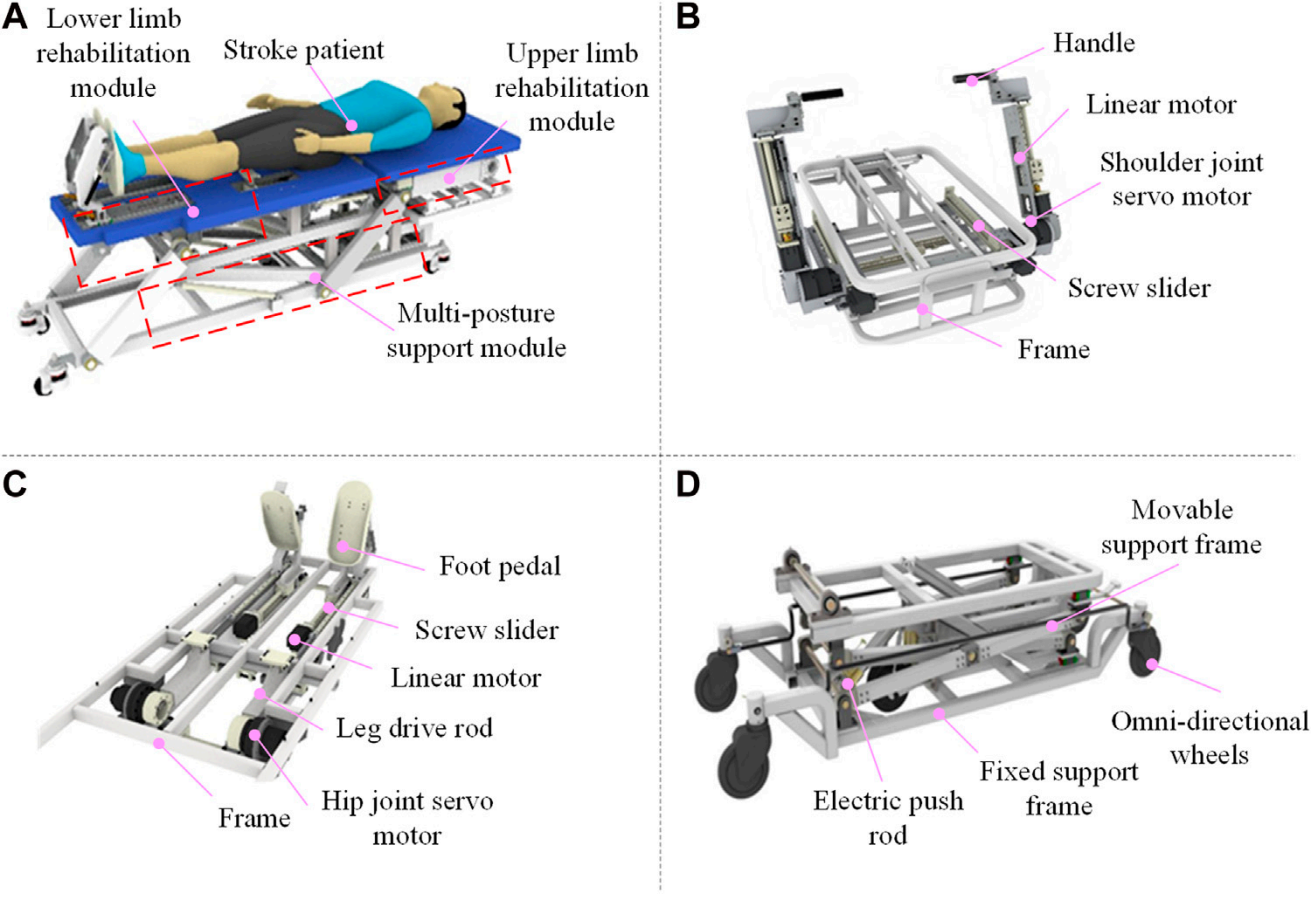
Structural diagram of the U-LLCRR. **(A)** Virtual overall prototype model. **(B)** Upper limb rehabilitation module. **(C)** Lower limb rehabilitation module. **(D)** Multi-posture support module.

Figure 1A depicts the structure and key components of the proposed U-LLCRR, including lower limb rehabilitation module, upper limb rehabilitation module, multi-postures support module. As depicted in Figure 1B, the upper limb rehabilitation module, comprising of the shoulder joint servo motor, linear motor, and handle, transmits the driving force from the shoulder joint motor to the patient’s whole upper limbs. The screw slider in the upper limb rehabilitation module adjusts the position of the shoulder joint servor motor to accommodate patients of varying heights. The linear motor induces linear motion at the wrist joint, enabling flexion and extension of bilateral shoulder-elbow-wrist joints in the human sagittal plane. In Figure 1C, the lower limb rehabilitation module adjusts the position of the ankle’s foot pedal by modifying the linear motor, accommodating patients of varying heights. The lower limb rehabilitation module connects to the patient’s thigh using velcro. The hip joint servo motor, leg drive rod, and foot pedal collaborate to transmit power from the servo motor to the patient’s thigh, This enables flexion and extension of bilateral hip-knee-ankle joints in the human sagittal plane. In Figure 1D, the multi-postures support module enables rehabilitation training for bedridden patients in different posture, including lying posture, inclined lying posture, and standing posture. The omni-directional wheels offer mobility and stability to the multi-postures U-LLCRR. The extension and retraction of the electric push rod could raise and lower the movable support frame, thereby altering the robot’s training postures’ height.

**FIGURE 2:**
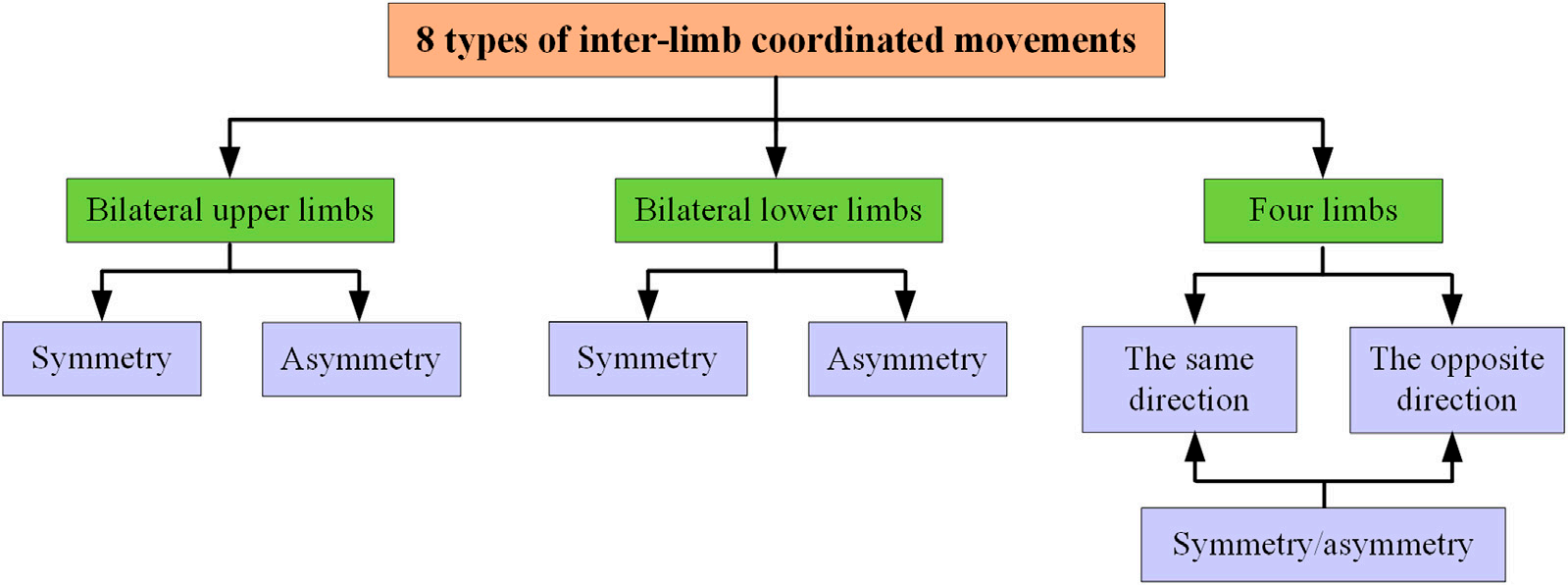
Eight types of inter-limb coordinated movements.

Based on the mechanism design of the proposed U-LLCRR, it can achieve 8 types of inter-limb coordinated movements, including bilateral upper limbs symmetry/asymmetry movement, bilateral lower limbs symmetry/asymmetry movement, limbs symmetry/asymmetry in the same direction movement, and limbs symmetry/asymmetry in the opposite direction movement (Figure 2). Among these, the same direction denotes that the upper and lower limbs on the same side move in the same direction, while symmetry implies that the left and right limbs move in the same direction. The proposed U-LLCRR overcomes the challenge of not being able to give patients synchronous upper and lower limb movements with current rehabilitation robots.

### 2.2 Hardware control system design of U-LLCRR

**FIGURE 3:**
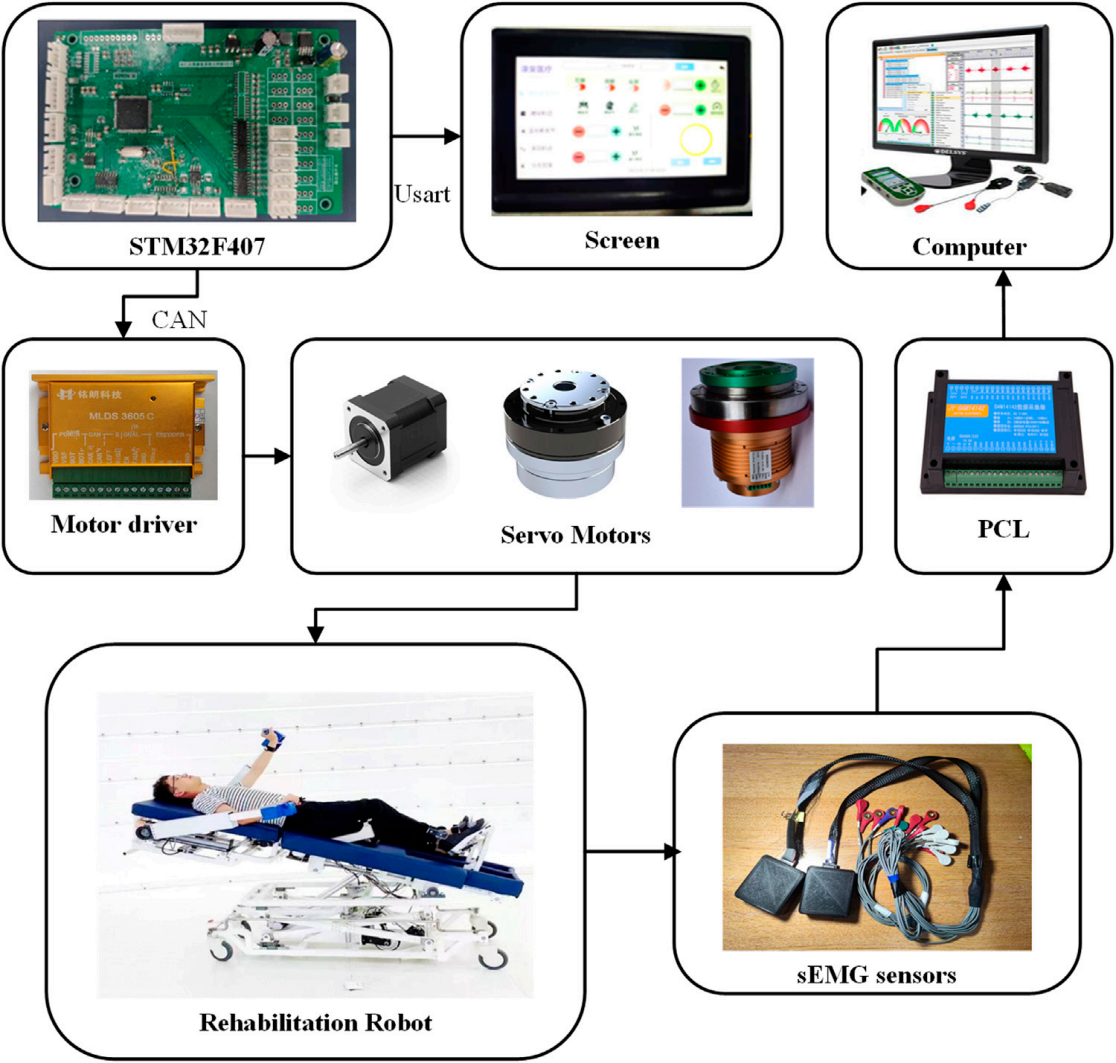
Electrical control and sEMG signal acquisition diagram for U-LLCRR.

The hardware control system of U-LLCRR utilises a distributed control structure, as seen in Figure 3. This hardware control system features a high-level medical serial screen coupled with a low-level STM32. The whole hardware control system comprises a serial screen, an embedded microcontroller, drivers, servo motors, and supplementary components. The STM32 development board establishes communication with the upper-level medical serial screen using a serial port operating at a baud rate of 115,200 bits per second. Additionally, it communicates with the motor driver using CAN protocol at a frequency of 1,000 Hz. Upon receiving inputs from the higher echelons, the STM32 employs motion decoding to produce control signals for the servo motors. This approach streamlines periodic rehabilitation training by guiding the patient’s limbs in continuous movements to predefined positions and velocities.

The study employs the BCIduino amplifier, developed by the Navigation Biology Company, for collecting sEMG data from specific muscle groups in the patient’s upper and lower limbs. The hardware of BCIduino amplifier is composed of 16-channel wireless sensors designed for the capture of sEMG signals. The system uses OpenBCI software to carry out real-time filtering and visualisation of the sEMG data. During rehabilitation robot training, electromyographic information is consistently collected from the patient and transmitted to the PC (ISK, Lenovo Inc.) for real-time analysis.

### 2.3 Selection of classification movements

**FIGURE 4:**
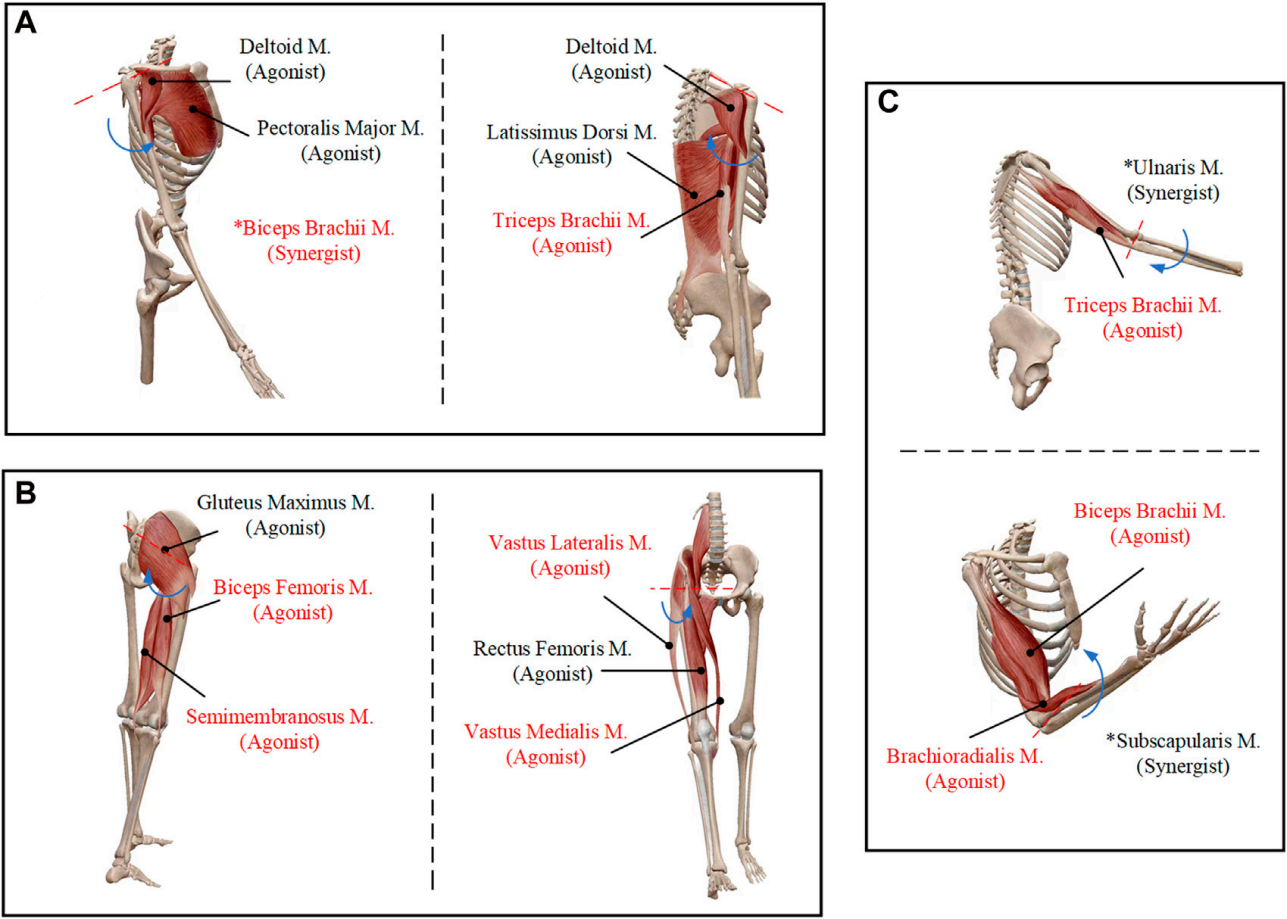
Six types of movements and the selected muscles. **(A)** Shoulder joint flexion/extension. **(B)** Elbow joint flexion/extension. **(C)** Hip joint flexion/extension.

This study defines a six particular limb movements which includes upper and lower limb movements of marching in place with arm swinging. And the defined movements could be realized through the proposed U-LLCRR. The activities mentioned relate to movements in the sagittal plane, specifically including the shoulder and elbow for the upper limbs, and the hip, knee and ankle for the lower limbs. The six types of limb movements consist of left arm shoulder joint flexion/extension (LS-FLX), left arm elbow joint flexion/extension (LE-FLX), right arm shoulder joint flexion/extension (RS-FLX), right arm elbow joint flexion/extension (RE-FLX), left leg hip joint flexion/extension (LH-FLX), and right leg hip joint flexion/extension (RH-FLX). Different limb motions are associated with different muscle groups, necessitating precise sensor positioning to capture signals. The process of associating various types of limb movements with specific muscle groups, while considering factors like ease of measurement, signals diversity, and accurate differentiation (Feng et al., 2021), led to decision to specifically target certain muscles in the upper and lower limbs as shown in Figure 4. The muscle groups highlighted in red font represent the specific muscles targeted for sEMG signal acquisition. For the upper limbs, the selected muscles are the deltoid, biceps brachii, triceps brachii, and brachioradialis. For the lower limbs, the chosen muscles are the biceps femoris, semitendinosus, adductor magnus, and tensor fasciae latae.

### 2.4 Evaluation of feature separability

Feature extraction is necessary for the 16-channel sEMG signals once they have been collected and preprocessed. The chosen features consist of four time-domain measures, like mean absolute value (MAV), root mean square (RMS), variance (VAR), and integrated EMG (iEMG), as well as two frequency-domain measures, like mean frequency (MF) and median power frequency (MPF). The prolonged and detrimental use of sEMG characteristics, which includes extraneous noise and interference from sensor cables, can result in reduced in computational speed and accuracy (Hu et al., 2021). This diminishes the system’s ability to recognise intentions in real-time. This study introduces a novel approach to selecting features in sEMG differing from existing methods. It presents a method for evaluating feature discriminability based on dispersion calculation.

For various limb movement classes, the separability of intra-class and inter-class distances is evaluated using the Fisher function as a discriminant criterion (Zhang et al., 2012). This method calculates the average separations between different feature vectors across various combinations. Initially, samples from various categories are projected onto a single dimension. Subsequently, the average separations between samples inside and between classes are computed. Following this, the ideal projection direction of the function is determined. The underlying concept is to maximize the average distance between classes while minimizing the average distance within them. This serves as the separability discriminant for different limb movement categories. In this study, the approach is further expanded to provide average distances between multiple classes.

$$\left\{\begin{array}{l} J_{d}\left(x\right)=\sum_{i=1}^{c}P_{i}\left[\frac{1}{n_{i}}\sum_{k=1}^{n_{i}}{\left(x_{k}^{\left(i\right)}-m_{i}\right)}^{T}\left(x_{k}^{\left(i\right)}-m_{i}\right)+{\left(m_{i}-m\right)}^{T}\left(m_{i}-m\right)\right]\\ m_{i}=\frac{1}{n_{i}}\sum_{k=1}^{n_{i}}x_{k}^{\left(i\right)}\\ m=\sum_{i=1}^{c}P_{i}m_{i} \end{array}\right.$$ (1)

In Eq. 1, $J_{d}\left(x\right)$ represents the distance between the D-dimensional sample of class *i* and samples from other classes. A larger value indicates better separability of the feature (Liu et al., 2013). $m_{i}$ is the mean vector of the i−th class sample set, m is the overall mean vector of all class sample sets, ${x_{k}}^{\left(i\right)}$ is the D-dimensional feature vector within class *i*, and $P_{i}$ is the prior probability of class *i*.

The Fisher fitness function, used in conjunction with the genetic algorithm, identifies the optimal combination of feature values. The algorithm iteratively determines the feature with the greatest dispersion among all features. The selected features form the input feature vector for the classification model.

### 2.5 Movement intention classification model

#### 2.5.1 Least Squares Support Vector Machine (LSSVM)

The LSSVM classification model is employed to classify the features that were extracted. LSSVM, an enhanced SVM algorithm (Mellit et al., 2012), is known for its rapid convergence, accuracy, and solution speed. To prevent the classifier from getting trapped in a local optimum and to enhance the predicted performance of the classification model, adjusting the parameters ‘*gam*’ and ‘*sig2*′ in LSSVM is crucial. Traditional methods for determining LSSVM settings often depend on historical performance data (Ahmad et al., 2014). This study enhances the classification performance of LSSVM by fine-tuning the parameters ‘*gam*’ and ‘*sig2*′ within predefined ranges using intelligent optimization algorithms (Xue and Shen, 2020).

#### 2.5.2 Improved BWOA based on sine chaotic mapping

Inspired by the hunting behavior of black widow spiders, characterized by both linear and spiral movements within their webs, the BWOA offers advantages in both local exploitation and global exploration (Hayyolalam and Pourhaji Kazem, 2019; Peña-Delgado et al., 2020). Population initialization, reproduction, intraspecific predation, mutation, and population update are its five stages. The remaining four stages, apart from the initial population stage, involve iteration until the termination criteria are met. This method employs LSSVM for classifying limb movements and determines the most fit “black widow” in the process.

The mathematical model is represented by Eq. 2:

$${\vec{x}}_{i}\left(t+1\right)=\left\{\begin{array}{cc} {\vec{x}}_{*}\left(t\right)-m{\vec{x}}_{r_{1}}\left(t\right), & \text{if}\;\text{rand}\left(\right)\leq 0.3\\ {\vec{x}}_{*}\left(t\right)-\cos\left(2\pi \beta \right){\vec{x}}_{i}\left(t\right), & \text{in}\;\text{other}\;\text{case} \end{array}\right.$$ (2)

In Eq. 2, ${\vec{x}}_{i}\left(t+1\right)$ denotes the updated individual position, ${\vec{x}}_{*}\left(t\right)$ represents the current optimal individual position, m is a random floating-point number generated between [0.4, 0.9], β is a random floating-point number within the range [-1, 1], $r_{1}$ is a random integer 1∼npop, ${\vec{x}}_{r_{1}}\left(t\right)$ signifies the randomly selected position at index $r_{1}$ , where $i\neq r_{1}$ , and ${\vec{x}}_{i}\left(t\right)$ is the current individual’s position.

The pheromone has a significant impact on the courtship behavior of black widow spiders. The pheromone deposition rate is defined as follows:

$$pheromone\left(i\right)=\frac{fitness_{\max}-fitness\left(i\right)}{fitness_{\max}-fitness_{\min}}$$ (3)

where, $fitness_{\max}$ and $fitness_{\min}$ denote the worst and best fitness values in the current population, respectively. $fitness\left(i\right)$ represents the fitness value of the i−th individual. And the pheromone vector contains fitness values normalized within [0,1].

Black widow spiders with low pheromone levels often resort to cannibalizing the female spiders of the same species. These individuals face collective rejection by the population and may be abandoned by the group (Houssein et al., 2020). During the iterative process, when an individual with low pheromone is abandoned, it becomes imperative to promptly replenish the population count. When *pheromone* is less than or equal to 0.3, the individual’s position is updated using Eq. 4:

$${\vec{x}}_{i}\left(t\right)={\vec{x}}_{*}\left(t\right)+\frac{1}{2}\left[{\vec{x}}_{r_{1}}\left(t\right)-{\left(-1\right)}^{\sigma }{\vec{x}}_{r_{2}}\left(t\right)\right]$$ (4)

where, ${\vec{x}}_{i}\left(t\right)$ represents the position of the black widow with low pheromone levels within the female’s body. $r_{1}$ and $r_{2}$ are random integers between 1∼npop, with $r_{1}\neq r_{2}$ ${\vec{x}}_{r_{1}}$ and ${\vec{x}}_{r_{2}}$ denote the positions of the black widow spiders at indices $r_{1}$ and $r_{2}$ , and $\sigma \in \left\{0,1\right\}$ is a random binary number.

Given that the initial positions of the black widow population are randomly generated, the study employs chaotic initialization using the Sine function from the chaos mapping strategy (Wu et al., 2021). This improves the quality of initial solutions, ensuring a more uniform distribution of the population within the search space. The expression is as follows:

$$\left.x_{k+1}=\frac{a}{n}\sin\left(\pi x_{k}\right),a\in (0,n\right]$$ (5)

Where k is the iteration count, $x_{k}$ is the k−th chaotic number, and a is a random number.

#### 2.5.3 Sine-BWOA-LSSVM classification model

**FIGURE 5:**
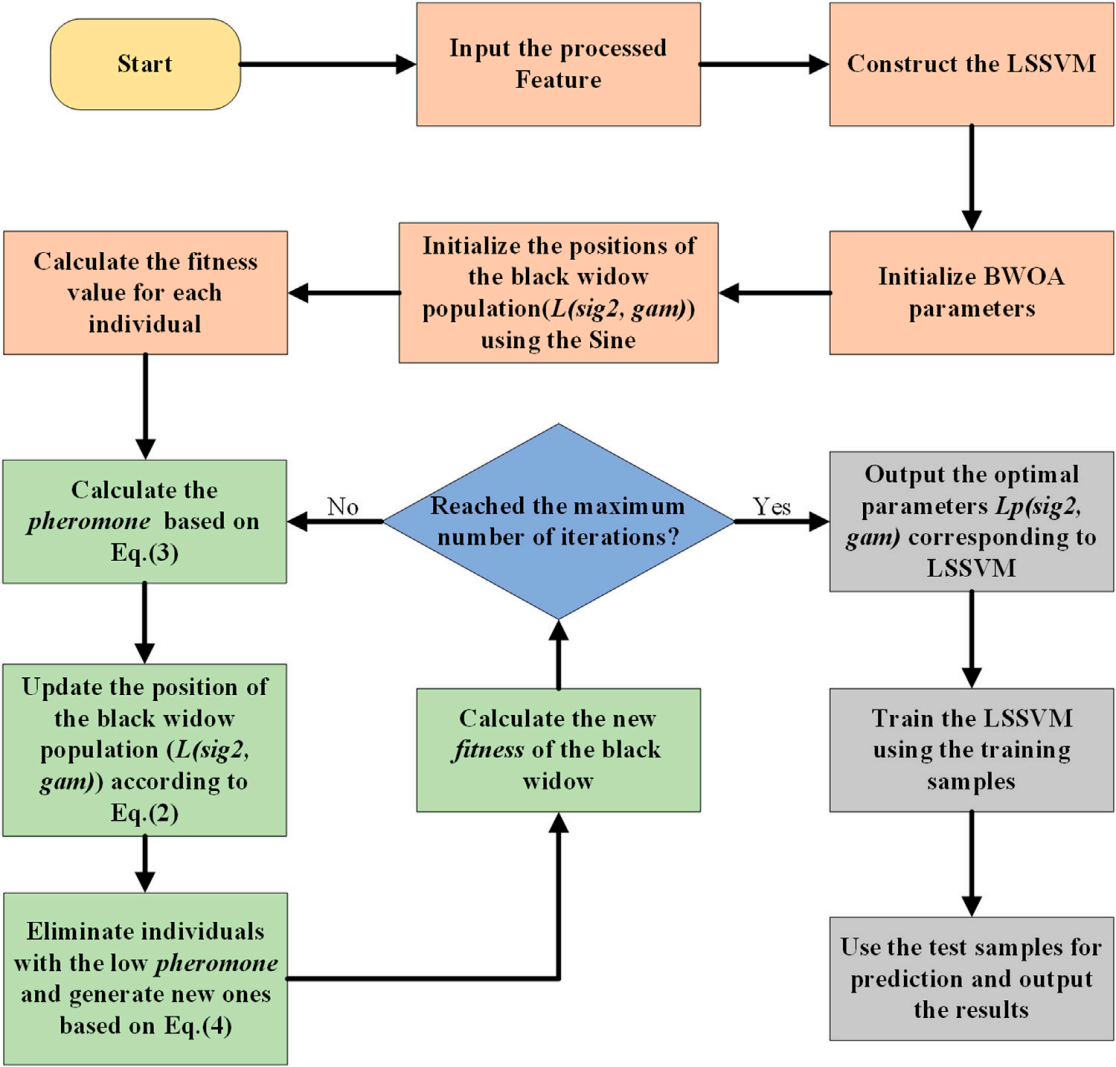
The SBL classification algorithm flow.

Each prediction model has its own set of advantages and disadvantages. By logically combining multiple single models, the shortcomings of each individual prediction model can be significantly mitigated, thereby enhancing forecast accuracy. To optimize the LSSVM classification method in Figure 5, this research presents a hybrid classification recognition model based on the improved BWOA with chaotic mapping, namely, the SBL classification model. The following are the precise steps:

• Step 1, Initialize basic parameters for BWOA, including the maximum number of iterations, procreating rate (PP), cannibalism rate (CR), and mutation rate (PM).
• Step 2, Initialize the positions of the black widow population using Sine chaotic mapping. The initial population is selected from fitness-sorted black widow individuals.
• Step 3, Use Sine-BWOA to optimize ‘*gam*’ and ‘*sig2*’ in LSSVM. Optimal parameters ‘*gam*’ and ‘*sig2*’ for LSSVM are obtained by iteratively updating the positions of black widow spiders.
• Step 4, Update LSSVM model, and conduct training and testing to obtain recognition results for feature output.

## 3 Experiments and results

Discrete limb combination experiments without robot assistance, and online decoding experiments based on the U-LLCRR are conducted as detailed in Table 1.

**TABLE 1:** Details of experimental protocol.

Experiment	Movement	Subject	Assistance	Data
I. Discrete test	Six types of limb movements	9	No	Online collection
II. Continuous decoding	‘Marching in place with arm swinging’ task	2	U-LLCRR	Online collection

### 3.1 Training and discrete testing of the SBL classifier

In this experiment, discrete data on six types of limb movements were collected from 9 healthy participants using a 16-channel sEMG signal capture device. Preprocessing procedures, such as noise reduction and bias removal, were applied to the collected sEMG signals. A **D** (96) feature vector, comprising six different types of features, was created from the extracted sEMG signal. In the feature selection process, a genetic algorithm and a discreteness computation were employed, resulting in **d** (48) feature vector. The data was split into a 30% test set and a 70% training set. Subsequently, the SBL classification model was trained offline using the training set. The trained model was subsequently applied for the recognition of online movements. To validate the robustness of the proposed categorization model, its experimental findings were compared with those from other models.

The subjects executed the aforementioned six types of limb movement combinations, performing each in 3 experimental sets. Each set consisted of 10 repetitions, with completion of each combination movement lasting approximately 4–5 s. The time interval between successive collections of the same combination movement was 6–8 s, and there was a 1–5 min interval between each set of experiments. Before each subsequent sEMG data collection session, it was confirmed that each subject was free from muscle fatigue. During the data collection process, the sensors continuously transmitted the acquired sEMG signals in real-time to a computer. This process yielded 180 data points for each type of limb movement per subject, resulting in a total of 2,880 data samples.

### 3.2 Continuous motion decoding

**FIGURE 6:**
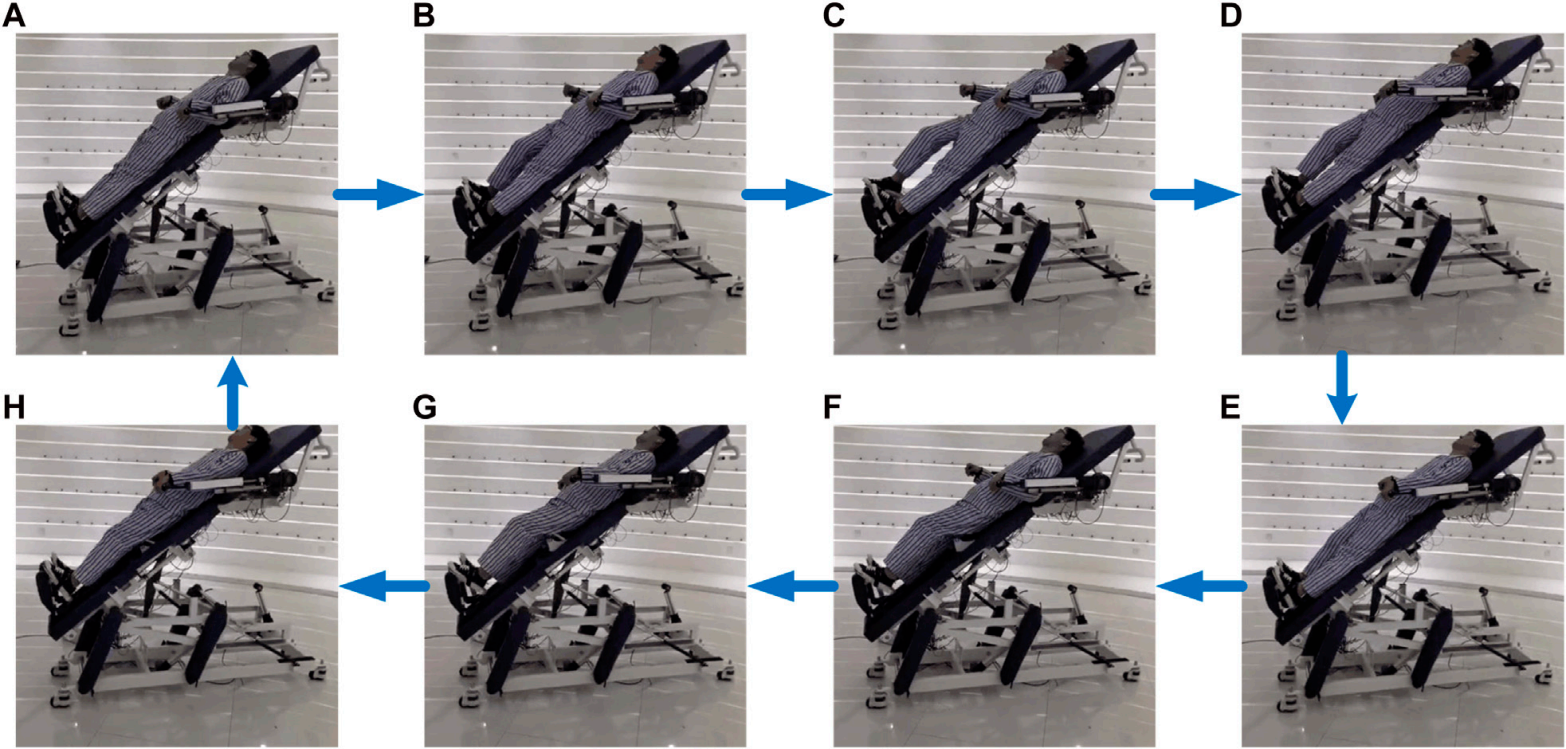
Actual upper and lower limbs coordinated movement process for subjects.

In order to verify the classification model’s ability to make accurate generalizations, a continuous motion recognition experiment is conducted, with the “Marching in place with arm swinging” assignment from the ADL training (Kwakkel et al., 2004). The experiment consists of four phases and fully includes the six types of selected limb movements mentioned above. The movement sequence is outlined in Table 2. Using the U-LLCRR, 2 subjects are selected to participate in the experiment. They perform the “Marching in place with arm swinging” continuously on the U-LLCRR, ensuring consistent and uninterrupted movement. Figure 6 illustrates the real-time identification of subjects’ sEMG data.

**TABLE 2:** Movement sequences in the ‘Marching in place with arm swinging’ task.

Order	Sub-movements	Time(s)	Order	Sub-movements	Time(s)
Phase 1			Phase 3		
1	LS-FLX	1–3	12	LE-FLX	34–36
2	LE-FLX	4–6	13	RS-FLX	37–39
3	RS-FLX	7–9	14	RE-FLX	40–42
4	RE-FLX	10–12	15	LH-FLX	43–45
5	LH-FLX	13–15	16	RH-FLX	46–48
6	RH-FLX	16–18	Phase 4		
Phase 2			17	LH-FLX	49–51
7	LH-FLX	19–21	18	RE-FLX	52–54
8	RE-FLX	22–24	19	RS-FLX	55–57
9	RS-FLX	25–27			
10	LE-FLX	28–30			
11	LS-FLX	31–33			

Each limb movement lasts for 3 s. Every subject performs four sets of continuous movements, following a specific motion sequence. Each set consists of 5 repetitions, totaling 20 repetitions per subject. The sEMG signals collected from subjects undergo the previously mentioned feature selection and SBL classification model method training. The training data is then input into the optimization algorithm for continuous limbs movement intention recognition, maintaining a 3:1 ratio for training and testing datasets, respectively. This process aims to validate the model’s accuracy in recognizing continuous movements and establishes a foundation for future research into the application of rehabilitation robots in active training.

### 3.3 Result of discrete test of the sine-BWOA-LSSVM classifier

#### 3.3.1 Feature processing

**FIGURE 7:**
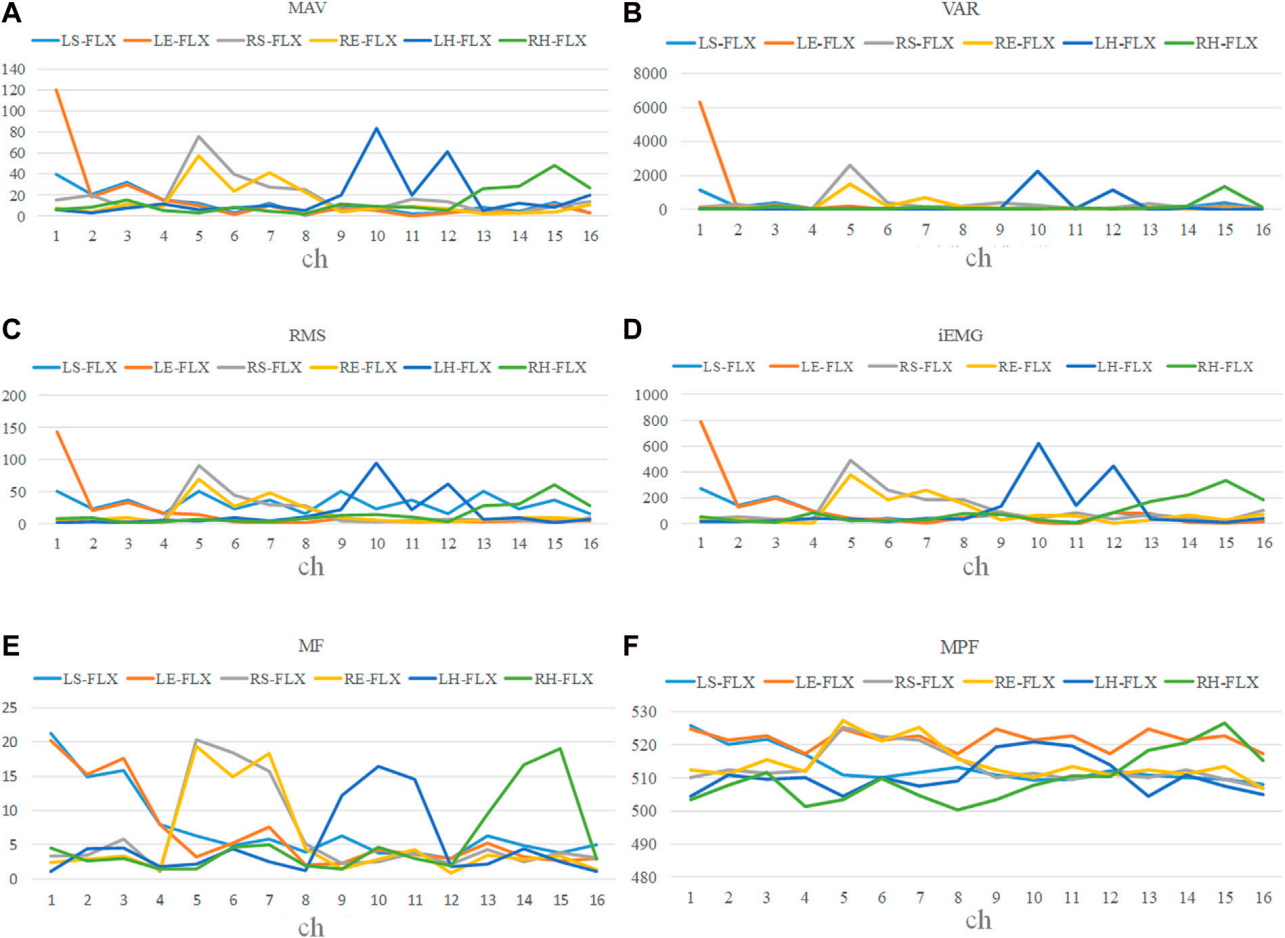
Frequency and time domain features for 16 channels.

After preprocessing the sEMG data obtained from 16 channels across 9 subjects, feature analysis was conducted in both the time and frequency domains. Figure 7 depicts the mean values of the six types of limb movements. The four time-domain features (MAV, RMS, VAR, iEMG) are somewhat effective in distinguishing between the movements. Some aspects demonstrated noticeable overlap, especially in movements involving the shoulder joint. Frequency-domain signals (MPF, MF) showed reduced variability and greater stability compared to the time-domain signals.

**FIGURE 8:**
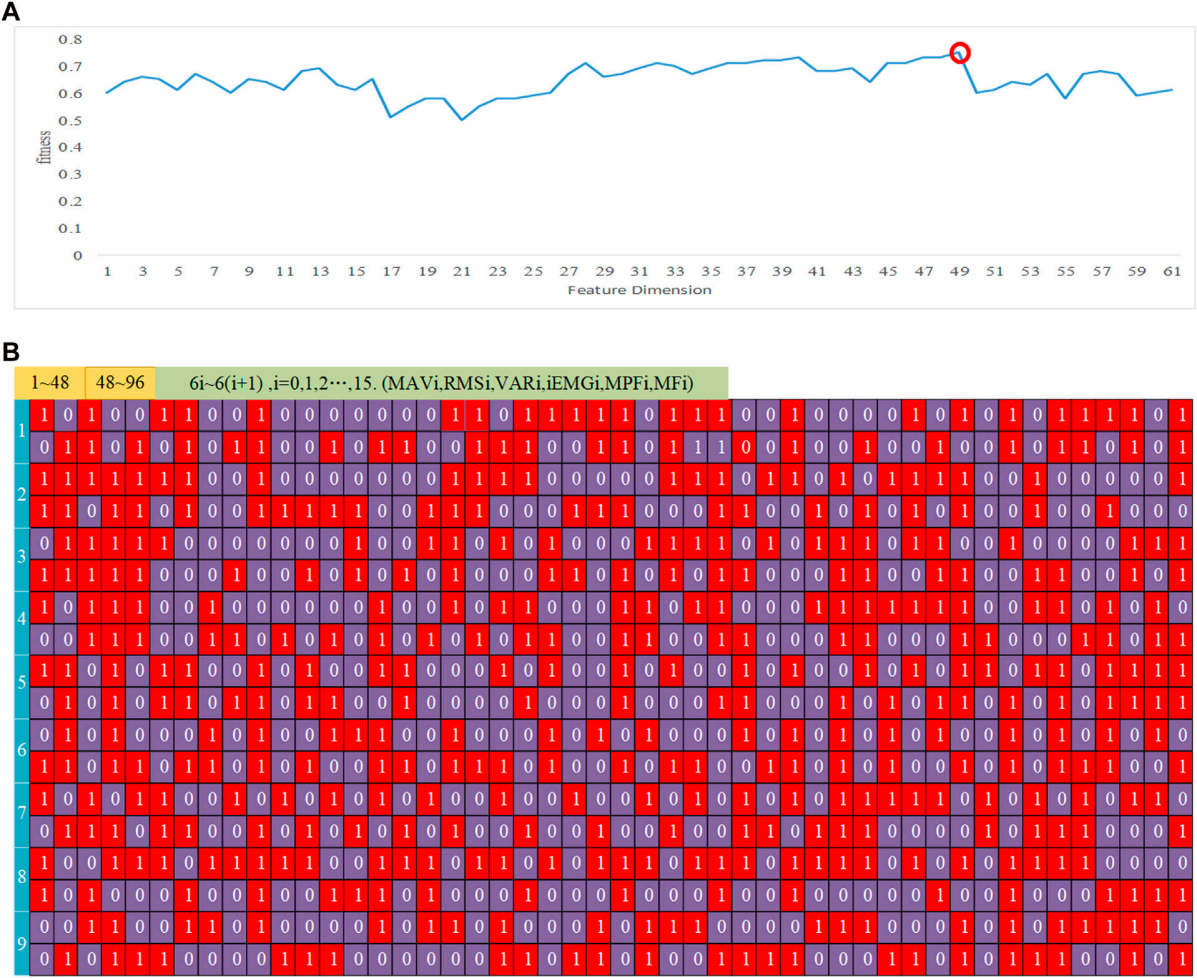
Optimal feature vector combinations. **(A)** Dissimilarity iteration curves across various dimensions and features. **(B)** The selection of optimal feature vectors.

Constructing larger-dimensional feature vectors by utilizing disparities among features enables more effective information extraction and improves movements differentiation. The calculated features were combined to form a 16 × 6 dimensional feature matrix encompassing various categories. To select a suitable multi-dimensional feature dimension, designated as **d** (*n*), dissimilarity was estimated using inter-class evaluation metrics from the above. Figure 8A illustrates the variations in dissimilarity across different dimensions and features after 100 cycles. Dissimilarity peaked at a feature dimension of 48, reaching a value of 0.75. Consequently, a total of 48 dimensions were selected. The 96-dimensional feature values were consolidated into **d** (48) composite feature vectors, and GA were repeatedly employed to assess dissimilarity using the Fisher function. Figure 8B displays the most favorable feature combinations of for the 9 subjects following iterative combination. The blue marks in Figure 8B represent the topic numbers, while the yellow on the right side indicates the layout. The red squares indicate a value of 1, representing the selected feature vectors, while the blue squares with a value of 0, represent the non-selected feature vectors.

#### 3.3.2 SBL classification model validation

In applying the classification model for recognizing limb movement intentions, the BWOA parameters were initialized with a population size (*Pop* = 20), the iteration index (*i* = [0:100]), *PP* = 0.8, *PM* = 0.4, and an infinitesimally small constant ( ε=10E−8 ).

**FIGURE 9:**
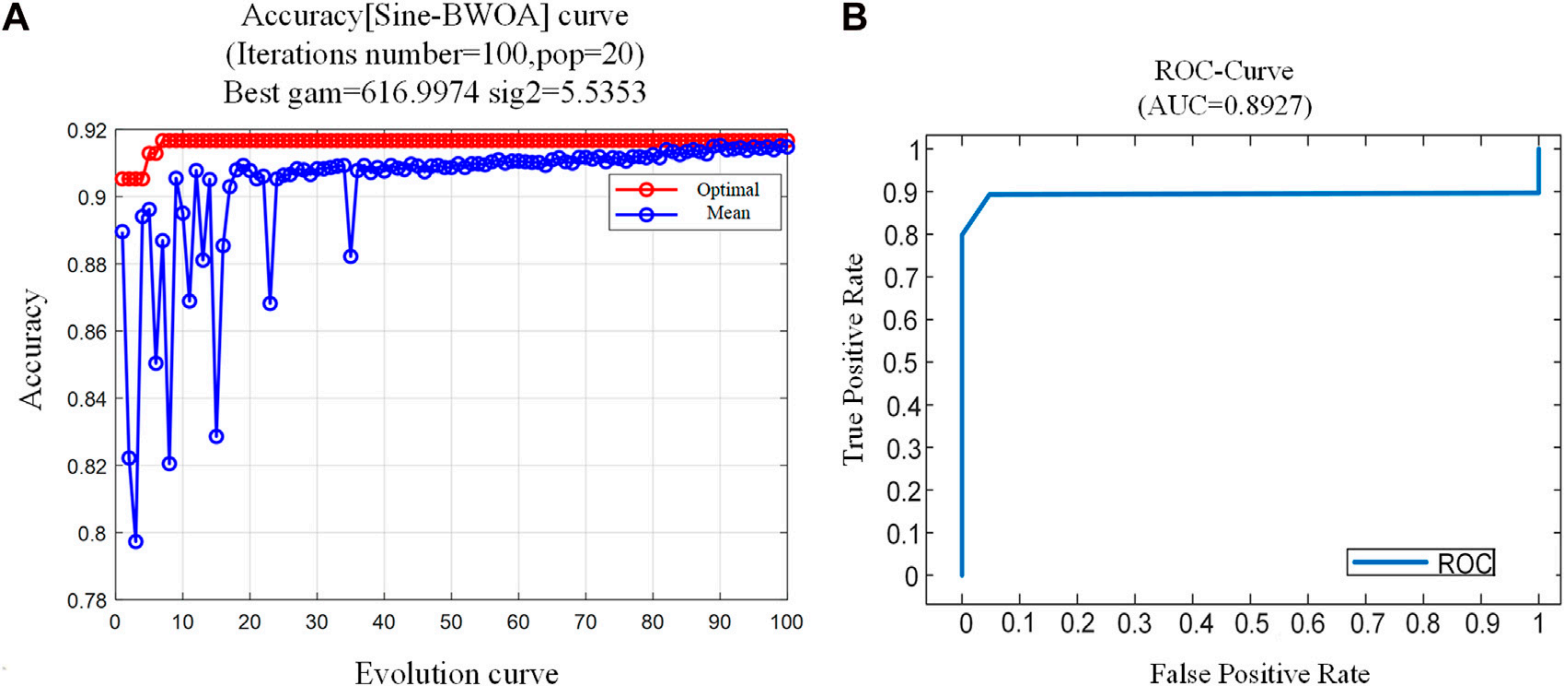
Training results of the SBL model. **(A)** Sine-BWOA fitness curve. **(B)** the ROC curve of the SBL model.

The first 15 instances of each movement, collected from the 9 subjects and totaling 1,440 instances, were used as the training dataset. The SBL classifier was trained using the optimal feature vectors obtained from the feature vector selection process (Figure 9), denoted as **d** (48) for each subject. Figure 9A shows the Sine-BWOA fitness variation curve, indicating that the population fitness gradually stabilizes after 35 iterations. The iterative results indicated that the optimal parameters for the LSSVM are *gam =* 616.9974 and *sig2 =* 5.5353. Figure 9B presents the ROC (receiver operating characteristic) curve for the model, with an AUC (area under curve) of 0.8927. Given that 1 > AUC > 0.5, it indicates that the SBL classification model exhibits good classification performance.

**FIGURE 10:**
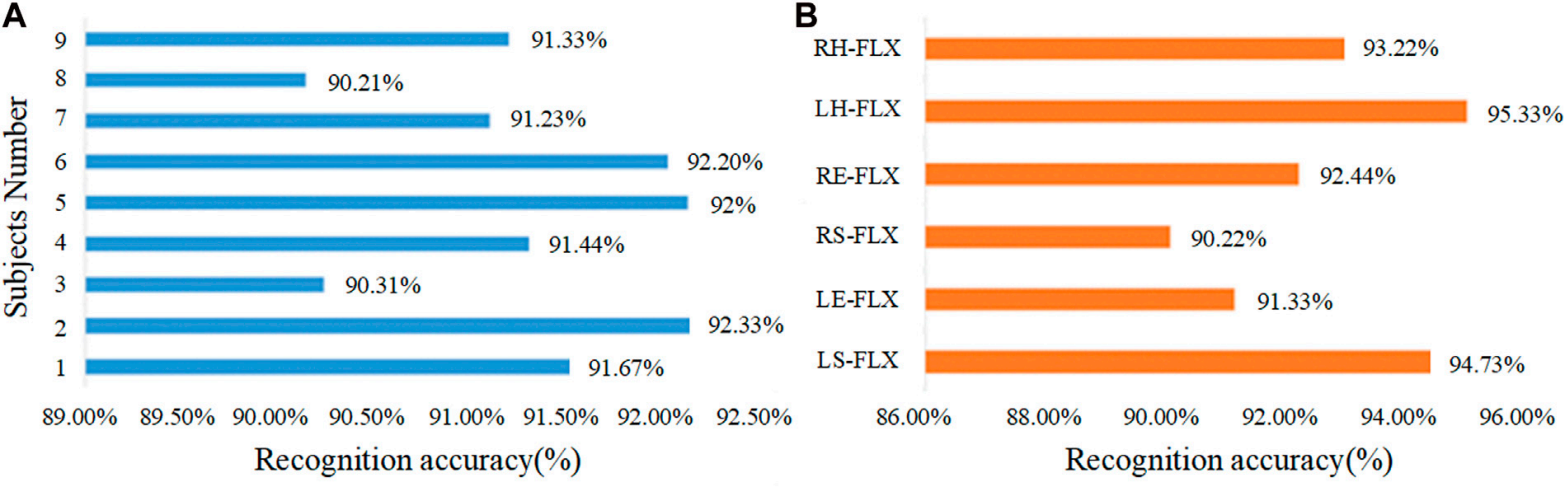
Accuracy of motion recognition **(A)** Subject body movements. **(B)** Discrete movements.

The SBL classification model was tested using the most recent 15 instances of each activity from 9 subjects, yielding a total of 1,440 instances. The outcomes of this test are illustrated in Figure 10A. The graph shows minor fluctuations in identification rates across the participants, but the overall results are quite consistent, with an average recognition rate of 91.413%. Subject No.8 has the lowest percentage of limb movement intention recognition rate at 90.21%, while the highest rate is 92.33% in Subject No.2. Figure 10B shows the results of the categorization test for the six types of limb movements, with an average recognition rate of 92.87%. Among the movements, LH-FLX has the highest recognition rate at 95.33%, while RS-FLX has the lowest recognition rate at 90.22%. The primary factor contributing to this variance is the susceptibility of sEMG signals from various muscle groups to interference during these specific actions.

**FIGURE 11:**
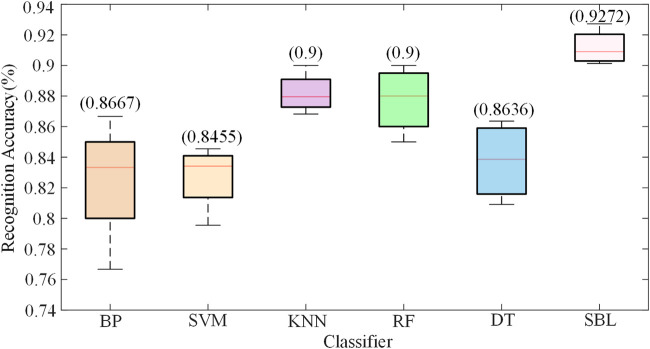
Recognition accuracy based on different classifiers.

To confirm the reliability of the developed the SBL classifier, the data were tested using five widely utilized classification algorithms, namely, SVM, BPNN (backpropagation neural network), KNN (k-nearest neighbor), RF (random forest), and DT (decision tree). Figure 11 provides a comparative analysis of the categorization performance. This figure clearly shows that the SBL classifier outperforms other classifiers in motion intention recognition, using the same sample data.

### 3.4 Result of continuous movement intentions decoding

**FIGURE 12:**
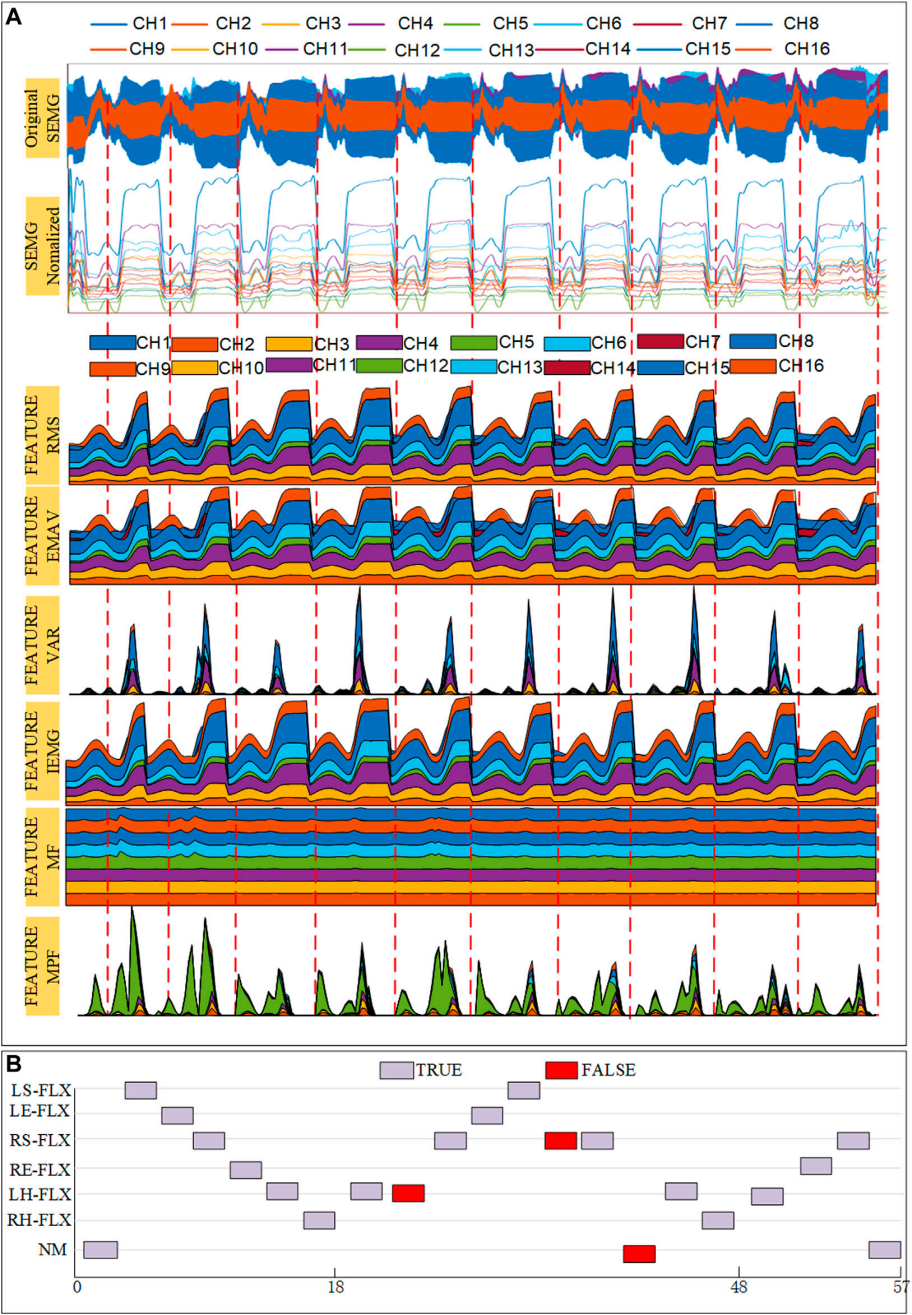
Experimental results of subject No. I in continuous movement. **(A)** Signal acquisition and feature value results **(B)** Continuous movement classification results.

During a specific instance of continuous movement, raw sEMG signals, normalized sEMG data, and an overlaid graph displaying characteristic values were gathered from subject No. I, as depicted in Figure 12A. The figure shows preprocessed data graphs derived from 16 channels of sEMG data, collected from both the upper and lower limbs. The data underwent denoising and normalization procedures. The overlaid graphs illustrate the extent of six characteristics measured from the processed data. The feature combination vectors were then inputted into the SBL classification model, and the resulting classification outcome is depicted in Figure 12B. In this depiction, ‘NM’ denotes the condition of no movement, grayscale squares represent accurate classifications, and red squares represent incorrect classifications.

Following the test findings, the confusion matrix for continuous limb movement recognition was derived, which was displayed in Table 3. In the table, the blue area indicates the count of accurate classifications, while the orange section indicates the count of incorrect classifications. This matrix presents the results of 240 consecutive movements performed by a single individual, detected using the SBL classifier. Subject No. I’s continuous movements achieved an average recognition rate of 89.25%. The recognition rates for the six types of limb movements RS-FLX, RE-FLX, and RH-FLX actions, are the highest, at 94.6%, 100%, and 95.29% respectively, all exceeding 90%. Conversely, LS-FLX, LE-FLX, and LH-FLX have recognition rates of 87.5%, 80%, and 87.5%, respectively. These rates fall within the 80%–90% range. The primary challenge contributing to this issue is the overlapping of movement processes, causing significant interference across muscle groups in different channels. Although continuous movement recognition may show reduced accuracy compared to individual movement intention recognition, the overall findings are still sufficiently reliable and suitable for subsequent active rehabilitation training using the U-LLCRR.

**TABLE 3:** Confusion matrix of recognition results.

movements to be recognized	Movements Recognition Results								Accuracy
		LS-FLX	LE-FLX	RS-FLX	RE-FLX	LH-FLX	RH-FLX	NM	
	LS-FLX	2	3						80.7%
	LE-FLX	1	20						95.2%
	RS-FLX			25		2			90.6%
	RE-FLX	1	1		17			1	89.47%
	LH-FLX					21	1		95.45%
	RH-FLX					1	26		95.29%
	NM	1		3		1		10	76.92%
Accuracy		87.5%	80%	94.6%	100%	87.5%	95.3%	90.9%	89.25%

## 4 Conclusion

A novel multi-postures upper and lower limb cooperative rehabilitation robot has been proposed, enabling the realization of eight distinct coordinated limb movements. This innovation establishes a physical platform for the identification of upper and lower limb coordinated movement intentions based on sEMG signals. Multi-dimensional sEMG signal classification and continuous movement recognition methods have been explored, leading to the proposal of a SBL classification model. It has been demonstrated through experimental results that the model excels in recognizing limb motion intentions, especially in differentiating between various limb movements, with some movement achieving recognition rates above 90%. Although a slight reduction in accuracy for continuous movement recognition has been observed, the overall results have been found to be reliable, rendering the model suitable for active rehabilitation training using the U-LLCRR. This outcome is significant for the development of more effective and personalized rehabilitation training programs. The effectiveness of the SBL model in continuous movement recognition has been validated, providing valuable insights for future advancements in rehabilitation technology. Future research will be directed towards enhancing the model’s robustness and exploring a broader range of movement patterns, with the aim of expanding the application scope of rehabilitation robot technology.

## Data Availability

The original contributions presented in the study are included in the article/Supplementary material, further inquiries can be directed to the corresponding authors.
